# Ultrasonic-assisted preparation of eucalyptus oil nanoemulsion: Process optimization, *in vitro* digestive stability, and anti-*Escherichia coli* activity

**DOI:** 10.1016/j.ultsonch.2021.105904

**Published:** 2021-12-29

**Authors:** Ruiteng Song, Yongqi Lin, Zhenzhen Li

**Affiliations:** a Department of Pharmacy, Affiliated Hospital of Weifang Medical University, Weifang, Shandong 261053, PR China; b School of Pharmacy, Weifang Medical University, Weifang, Shandong 261053, PR China

**Keywords:** Anti-*E. coli* activity, Eucalyptus oil, Nanoemulsion, Response surface methodology, Simulated digestion, Ultrasonic

## Abstract

Eucalyptus oil (EO) is a natural and effective antimicrobial agent; however, it has disadvantages such as poor water solubility and instability. The aim of this study was to investigate the effect of process vessels and preparation process parameters on the particle size of the emulsion droplets using ultrasonic technique and response surface methodology to prepare eucalyptus oil nanoemulsion (EONE). The optimal sonication process parameters in conical centrifuge tubes were confirmed: sonication distance of 0.9 cm, sonication amplitude of 18%, and sonication time of 2 min. Under these conditions, the particle size of EONE was 18.96 ± 4.66 nm, the polydispersity index was 0.39 ± 0.09, and the zeta potential was −31.17 ± 2.15 mV. In addition, the changes in particle size, potential, micromorphology, and anti-*Escherichia coli* activity of EONE during digestion were investigated by *in vitro* simulated digestion. The emulsion was stable in simulated salivary fluid, tended to aggregate in simulated gastric fluid, and increased in particle size and potential value in simulated intestinal fluid. EONE showed higher anti-*E. coli* activity than EO by simulated digestion. These results provide a useful reference for the *in vivo* antimicrobial application of the essential oil.

## Graphical abstract

**Figure:**
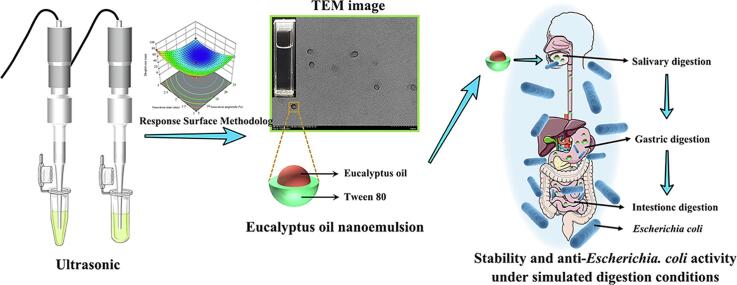

## 1 Introduction

*Escherichia coli* (*E. coli*) is one of the most common foodborne pathogens that causes gastrointestinal disease in humans and has the risk of causing sepsis [1], [2]. In recent years, several outbreaks of *E. coli* have occurred in developed countries, posing a serious threat to the public health safety of the population [3], [4]. Antibiotics are commonly used clinically to treat *E. coli* infections [5], [6]. However, the global problem of drug resistance due to antibiotic abuse has prompted a continuous search for new antimicrobial agents that are safe, natural, and efficient.

The antimicrobial activity of plant essential oils *in vitro* and *in vivo* has attracted great interest [7], [8]. The main components of eucalyptus oil (EO) are terpenoids and alcohols with antimicrobial, anti-inflammatory, and hypolipidemic activities [9], [10], [11]. EO has been generally recognized as a safe substance by the US Food and Drug Administration due to its safe, efficient, and nontoxic characteristics, and is widely used in pharmaceutical, industrial, and food applications [12]. The national standard of Chinese food additives GB2760-2014 stipulates that EO (N114) should be used appropriately in all kinds of food according to the needs of production. According to the survey of the Flavor and Extract Manufacturers Association (FEMA), the *per capita* intake of EO (FEMA 2466) was 260 μg/(person ⋅ day) [12]。Compared with the use in over-the-counter drugs, EO is used at much lower concentrations as a flavoring ingredient in foods, ranging from approximately 4 to 2000 ppm. Eisenbrand *et al.* [12], based on the *per capita* intake of EO and no observed adverse effects of EO on rats [300 mg/(kg bw ⋅ day)], showed that the margin of safety of EO was greater than 69,000. We intended to use EO as an antibacterial substance and set the concentration of EO to 1% to retain the antibacterial activity of EO against *E. coli*. However, the poor water solubility, volatility, and short efficacy of EO limited its application.

A nanoemulsion is a kinetically stable dispersion system of small droplets dispersed in another immiscible liquid, forming droplets with a particle size <200 nm. Nanoemulsions improve the bioavailability and absorption of lipophilic compounds and can be used as an effective delivery system for various lipophilic compounds. They can enhance the antimicrobial effect by increasing the contact area of active ingredients and overcoming the low permeability of bacterial cell membranes [13], [14], [15], [16].

Low-energy preparation of nanoemulsions requires high concentrations of surfactants, leading to toxicity [17]. The ultrasonic emulsification method not only is efficient and economical but also reduces the amount of emulsifier and yields smaller particle size; hence, it is widely used by researchers [18], [19]. Ultrasonication can also be used to adjust some properties of nanoemulsions, such as stability, particle size, polydispersity index (PDI), and zeta potential, by changing process parameters. It generates strong turbulence and micro-jets through cavitation to break large droplets into small ones [20]. Response surface methodology (RSM) is a statistical and mathematical approach using quadratic polynomial models to study the relationship between one or more response variables and several independent variables [21], [22]. Univariate models, though simpler, are time-consuming and lack the influence of interrelationships between independent variables in optimization [23]. Box-Behnken design (BBD) is one of the multivariate optimization methods for quadratic response surface models [24]. The experimental design is simple and efficient, saving cost and time [25].

In this study, RSM was used to optimize the three process parameters of sonication distance (distance from the bottom of the vessel to the ultrasound probe), sonication amplitude, and sonication time, and to screen a suitable preparation vessel to prepare O/W-type EONE with small droplet size and more uniform particle size distribution so as to improve its bioavailability and antibacterial activity. The anti-digestive ability of EONE and the antibacterial activity of each simulated digestion stage were further investigated to provide some research basis for the *in vivo* antibacterial application of the essential oil.

## 2 Materials and methods

### 2.1 Materials

EO, Tween 80, glutaraldehyde, sodium chloride, potassium chloride, mucin (II), potassium dihydrogen phosphate, ammonium nitrate, ammonium chloride, pepsin, trypsin, and sodium hydroxide were purchased from Shanghai Maclean Bioreagent Co. Ltd. (Shanghai, China). Propidium iodide (PI) was purchased from Beijing Solarbio Science Technology Co. Ltd. (Beijing, China)*. E. coli* ATCC 11,229 strain was obtained from the College of Pharmacy, Weifang Medical College.

### 2.2 Ultrasonic-assisted preparation of EO nanoemulsion

#### 2.2.1 Preparation of crude emulsion

**Fig. 1:**
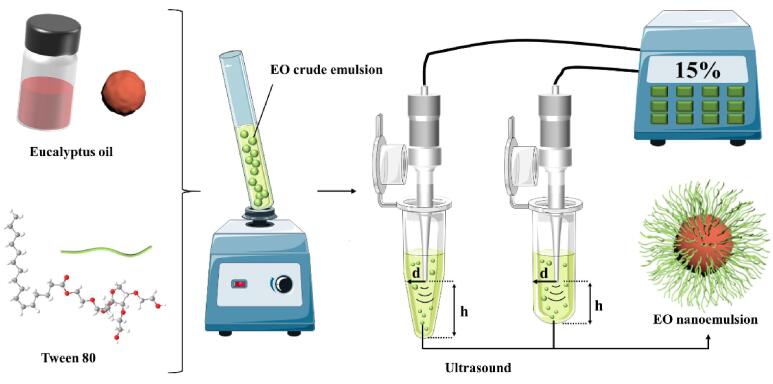
Schematic diagram of the eucalyptus oil (EO) nanoemulsion preparation.

We prepared crude emulsion by the previously described method [26]. As shown in Fig. 1, EO emulsions were prepared by mixing oil phase, water phase, and emulsifier. The oil phase was EO, the water phase was distilled water, and the emulsifier was Tween 80. 496 mg Tween 80 was dissolved in 49 mL of distilled water. Then, 4.95 mL of the aqueous phase was slowly added to 0.05 mL of the oil phase at room temperature (25 °C) and shaken for 3 min on a vortex mixer (QL-901, China Aomen) to obtain the crude emulsion. The concentration of EO in the crude emulsion was 1.0% (*v*/*v*), and the concentration of Tween 80 was also 1.0% (*w*/*w*).

#### 2.2.2 Selection of the ultrasonic vessel

As shown in Fig. 1, the crude emulsion was sonicated to obtain emulsions with smaller droplet sizes and more uniform particle size distribution. An 750 W ultrasonic cell crusher (Scientz-II D; Ningbo Scientz, Zhejiang, China) with a frequency of 20 kHz was used to ultrasonicate 1.0 mL of the crude emulsion in the vessels to prepare nanoemulsions. The vessels could be either a 1.5-mL conical centrifuge tube or a 2.0-mL round-bottom centrifuge tube. The ultrasound probe tip diameter was 3 mm, and the thickness of the vessels was 0.72 ± 0.11 mm. The ultrasound operational conditions were as follows: the height (h) from vessel bottom to ultrasound probe tip; horizontal position of the ultrasound probe (d): at the center, 2.40–4.59 mm away from the center, sonication time: 1–3 min, and sonication amplitude: 5%–25% (sonication power: 37.5–187.5 W) displayed as the power level on the device. The time interval was 5 s, and the vessels were placed in an ice water bath during the sonication process. After sonication, the samples were stored at 25 °C and protected from light.

#### 2.2.3 Optimization of ultrasound parameters using RSM

RSM is widely used for ultrasonic process optimization because of the effectiveness of its mathematical model fitting. Pongsumpun *et al.* [23] optimized sonication time (sec), temperature (°C), and Tween 80 (%) using BBD. Ahmed *et al.* [27] optimized BBD of oil (%), smix (%), and sonication time. In this study, the sonication distance (*X*_1_), sonication amplitude (*X*_2_), and sonication time (*X*_3_) were investigated using a three-factor, three-level BBD (Table 1) to examine the effects of each variable on the particle size. The procedure was performed 17 times, while the center point was repeated 5 times. In this study, the values of variables were as follows: sonication distance: 0.9–1.5 cm; sonication amplitude: 5%–25% (sonication power: 37.5–187.5 W), and sonication time: 1–3 min. The sonication parameters were optimized with the goal of reducing the droplet particle size and PDI. The independent variables were coded with values between −1 and 1. All independent variables were run at three levels according to BBD (Table 1) for each individually coded value (1, 0, and 1). The BBD design was used to explore the effects of the independent variables and the interactions between the independent variables on the response variables. This effect could be expressed as Eq. (1)

(1) $$Y_{i}=b_{0}+b_{1}x_{1}+b_{2}x_{2}+b_{3}x_{3}+b_{11}x_{1}^{2}+b_{12}x_{2}^{2}+b_{13}x_{3}^{2}+b_{21}x_{1}x_{2}+b_{22}x_{1}x_{3}+b_{23}x_{2}x_{3}$$

where *Y* is the response variable; *b*_0_ is the constant in the equation; *b*_1_, *b*_2_, and *b*_3_ are the linear regression coefficients; *b*_11_, *b*_12_, and *b*_13_ are the quadratic regression coefficients; and *b*_21_, *b*_22_, and *b*_23_ are the independent variable interaction regression coefficients. The coded values of the four parameters (*x*_i_) could be obtained from Eq. (2).

(2) $$x_{i}=\left(x_{i}-x_{0}\right)/\Delta X$$

where *x_i_* is the actual value of the dependent variable, *x*_0_ is the actual value of the dependent variable at the center point, and ΔX is the value of the horizontal change in the dependent variable. A quadratic polynomial equation Eq. (1) was fitted to the data obtained from 17 trials in BBD using Design expert (version 11.1.0.1, Stat-Ease, MN, USA), and analysis of variance (ANOVA) was performed to test the statistical significance of the equation. The corresponding 3D response surface plots were also drawn. All experiments were performed three times in parallel.

**Table 1:** Box-Behnken design with natural and coded ultrasonication conditions.

Std	Sonication distance (*X*_1_)	Sonication amplitude (*X*_2_)	Sonication time (*X*_3_)	Sonication power
1	0.9 (–1)	5 (–1)	2 (0)	37.5
2	1.5 (1)	5 (–1)	2 (0)	37.5
3	0.9 (–1)	25 (1)	2 (0)	187.5
4	1.5 (1)	25 (1)	2 (0)	187.5
5	0.9 (–1)	15 (0)	1 (–1)	112.5
6	1.5 (1)	15 (0)	1 (–1)	112.5
7	0.9 (–1)	15 (0)	3 (1)	112.5
8	1.5 (1)	15 (0)	3 (1)	112.5
9	1.2 (0)	5 (–1)	1 (–1)	37.5
10	1.2 (0)	25 (1)	1 (–1)	187.5
11	1.2 (0)	5 (–1)	3 (1)	37.5
12	1.2 (0)	25 (1)	3 (1)	187.5
13	1.2 (0)	15 (0)	2 (0)	112.5
14	1.2 (0)	15 (0)	2 (0)	112.5
15	1.2 (0)	15 (0)	2 (0)	112.5
16	1.2 (0)	15 (0)	2 (0)	112.5
17	1.2 (0)	15 (0)	2 (0)	112.5

### 2.3 EONE particle size, PDI, zeta potential, and micromorphology

The particle size distribution, PDI, and zeta potential of EONE were determined using a Malvern dynamic light scattering instrument Zetasizer (Nano ZS90, Malven Instruments Ltd., UK) [28]. The samples were diluted with double distilled water (1:100, *v*/*v*) before measurement to avoid multiple scattering effects [29], [30]. Afterward, EONE was mounted on a carbon-coated grid for negative staining with 2.0% phosphotungstic acid for 30 min. After air drying, the samples were observed and photographed with a transmission electron microscope (TEM, Hitachi, HT7700, Japan).

### 2.4 Encapsulation efficiency

The encapsulation efficiency (EE) of EONE was determined by the ultracentrifugation method. We referred to the method of Zhou *et al.* [31] to make appropriate modifications. Briefly, 3.0 mL of the sample was mixed with 5.0 mL of distilled n-hexane, and EO was extracted with n-hexane in a water bath at 50 °C. EO was centrifuged at 12,000*g* for 15 min using a high-speed refrigerated centrifuge (3–30 k, Sigma, Germany). The free EO content in *n*-ethanol was measured using an ultraviolet spectrophotometer (UV-800a, Shanghai Yuanxi Instrument Co., Ltd., China) at the maximum absorption wavelength of 206 nm, and the EO content was calculated by substituting into the standard curve. The standard curve was plotted using different concentrations of EO in ethanol (ranging from 2.5 to 20 nL/mL ethanol) with equation *A* = 0.0307*x* (concentration of EO as nL/mL ethanol) + 0.0154 (*R*^2^ = 0.9934). The EE equation is expressed as Eq. (3).

(3) $$EE\left(\%\right)=\left(1-F/I\right)\times 100\%$$

where “*F*” is the EO content in n-hexane, and “*I*” is the EO content added during emulsion preparation.

### 2.5 Establishment of *in vitro* digestion model

A three-stage (oral, gastric, and intestinal) *in vitro* digestion model was established based on previous reports with slight modifications [32], [33], [34]. The first step was the preparation of simulated digestive juices for each digestion stage. The simulated salivary fluid (SSF) contained 1.594 g/L NaCl, 0.202 g/L KCl, 10 g/L mucin (II), 0.638 g/L potassium phosphate, and 0.328 g/L ammonium nitrate. The simulated gastric fluid (SGF) contained 2.0 g/L ammonium chloride, 7 mL/L hydrochloric acid, and 3.2 g/L pepsin. The simulated intestinal fluid (SIF) contained 10 g/L pancreatic enzyme and 0.05 mol/L potassium dihydrogen phosphate, pH 7.4 (adjusted with sodium hydroxide). Each digestion solution was preheated in a water bath at 37 °C for 10 min before performing the digestion test.

Furthermore, 10 mL of EONE was incubated with 5 mL of SSF at 37 °C for 10 min at a spinning speed of 100 rpm, transferred to 10 mL of SGF, and incubated at 250 rpm for 1 h. Finally, EONE was transferred to 10 mL of SIF and incubated for 2 h at 37 °C at 250 rpm. During SSG, SGF, and SIF incubation, 500 μL of the samples were removed at predetermined time intervals and mixed with 100 μL of sodium hydroxide (2.5 M) to stop the enzymatic reaction and proceed to the next step.

### 2.6 Measurement of changes in EONE during digestion

The droplet size, PDI, zeta potential, and micromorphological changes in the samples during digestion were measured as described in Method section “2.3.”

### 2.7 Antimicrobial activity of EONE in each digestion stage

#### 2.7.1 Minimal inhibitory concentration and minimal bactericidal concentration

To investigate the anti-*E. coli* effect of EONE in each digestion solution, minimal inhibitory concentration (MIC) and minimal bactericidal concentration (MBC) were determined by the multiplicative dilution method [26], [35]. Single colonies of *E. coli* were inoculated in Luria-Bertani liquid medium, incubated at 37 °C for 18 h at 180 rpm, and then centrifuged at 5000*g* for 5 min. The bacteria at the bottom were washed three times with phosphate-buffered saline (PBS) solution, and the bacterial concentration was adjusted to 2.0 × 10^5^ CFU/mL with LB liquid medium. Sterile saline was used for the blank control group, and the experimental groups were EO, EONE, SSF (simulated salivary fluid digestion for 10 min), SGF (simulated gastric juice digestion for 1 h), and SIF groups (simulated intestinal juice digestion for 2 h). Nine tubes with sterilized LB liquid medium were prepared for each group, and samples were added to each tube to achieve final concentrations of 2.56, 1.28, 0.64, 0.32, 0.16, 0.08, 0.04, 0.02, and 0.01 µL/mL of essential oil; also, *E. coli* bacterial solution was added to achieve a concentration of 1.0 × 10^5^ CFU/mL per tube. The bacteria were incubated at 37 °C and centrifuged at 180 rpm for 18 h. Each bacterial suspension was transferred to a 96-well plate, and the OD_600_ was measured with a multifunctional microplate reader (SpectraMax M5; Molecular Devices, CA, USA) using an uninoculated LB liquid medium as the zeroing point. The bacterial suspensions with high cell density were appropriately diluted with LB liquid medium and then measured so that the OD_600_ values were in the range of 0.1–0.65; the process was repeated three times. The lowest concentration that allowed bacterial growth to be inhibited and the bacterial solution to remain clarified was determined as MIC by data and appearance analyses. The OD_600_ values in each group were substituted into Eq. (4).

(4) $$\mathrm{Inhibition}\;\left(\%\right)=\left(1\;-\;T/C\right)\;\times \;100\%$$

where “*T*” is the OD_600_ value in the samples in each experimental group, and “*C”* is the OD_600_ value in the blank control group; each group was parallelly repeated three times. The MIC of EONE against *E. coli* was used as the lowest concentration. Then, 10 μL of the bacterial solution was applied to LB solid medium from each group of test tubes, placed in a constant-temperature incubator, and incubated upside down for 24 h at 37 °C. The number of single colonies in the Petri dishes was calculated, and the lowest concentration at which no single colony appeared in each experimental group was the MBC.

#### 2.7.2 Bacterial inhibition kinetics

The antimicrobial activity of EONE was studied by monitoring the change in colony count over time in each digestion stage [36]. After *E. coli* was cultured to the log phase and diluted to 5 × 10^6^ CFU/mL, the control group (saline group) and each experiment group (EONE, EO, SSF, SGF, and SIF groups) containing essential oil (2.56 μg/mL) were mixed with the bacterial solution individually in sterile test tubes and incubated at 37 °C in a shaker at 180 rpm. The number of colonies at different time points was recorded by the colony counting method.

#### 2.7.3 Scanning electron microscopy

Scanning electron microscopy (SEM) was performed to observe the changes in EONE on *E. coli* cell morphology in each digestion stage. We referred to the method of Wu *et al.* [35] with appropriate modifications. The bacterial suspensions from each group of treatments were centrifuged at 8000 rpm for 5 min at 4 °C. The supernatant was discarded, and the bacteria were washed with PBS buffer two times. After fixing the bacteria with 2.5% glutaraldehyde for 12 h, gradient alcohol dehydration was performed. The cells were dried and sprayed with gold plasma. The samples were observed using an SEM (JSM-840, Jeol, Japan).

#### 2.7.4 Bacterial integrity test

The integrity of *E. coli* cell membranes was assessed using PI fluorescence staining according to the previously reported method [26]. The experiment was divided into five groups: blank control group (saline), EO group, EONE group, SSF group, SGF group, and SIF group. *E. coli* was cultured to 1.0 × 10^7^ CFU/mL, repeatedly mixed with samples from each group, and incubated at 180 rpm for 2 h at 37 °C. The supernatant was then removed by centrifugation at 5000*g* for 5 min at 4 °C, and the bacteria were diluted with PBS buffer; the process was repeated three times. Then, 200 μL of PI staining solution was added, incubated for 20 min at room temperature in the dark, and centrifuged at 5000*g* for 5 min. The supernatant was discarded, and PBS was added. The bacteria were blown gently, centrifuged at 5000 *g* for 5 min, and diluted with PBS. Further, 10 μL of the bacterial solution was added dropwise on a slide. The bacteria were observed and photographed at 535 nm using a fluorescence microscope (Leica DM4B, Germany), and the number of bacteria was calculated using Image J 1.48v (National Institutes of Health, MD, USA).

#### 2.7.5 Detection of bacterial nucleic acids *in vitro*

We refer to the method of Shu *et al* [37] to make appropriate modifications. EO, EONE, SSF, SGF, and SIF solutions were added individually to 5 mL of 1.0 × 10^6^ CFU/mL of *E. coli* bacterial solution. After mixing well, the EO content of each group was controlled at 2.56 µL/mL. The blank control was the bacterial suspension without sample. The mixture was further incubated at 37 °C in a shaker at 180 rpm for 2 h. The supernatant was filtered through a 0.22-μm membrane and centrifuged at 8000 rpm for 3 min. The filtrate at the treatment time of 0 min for each group was set as control, and the concentrations of nucleic acids and proteins in the supernatant were determined by UV spectrophotometry at 260 nm and 280 nm, respectively.

### 2.8 Statistical analysis

Each experiment was conducted in triplicate, and the results were expressed as mean ± standard deviation. One-way analysis of variance was performed on the data using SPSS. Differences between groups were considered significant at the *P* < 0.05 level and plotted using Origin Pro 2019 (OriginLab Corporation, MA, USA).

## 3 Results

### 3.1 Screening of ultrasonic vessels

**Fig. 2:**
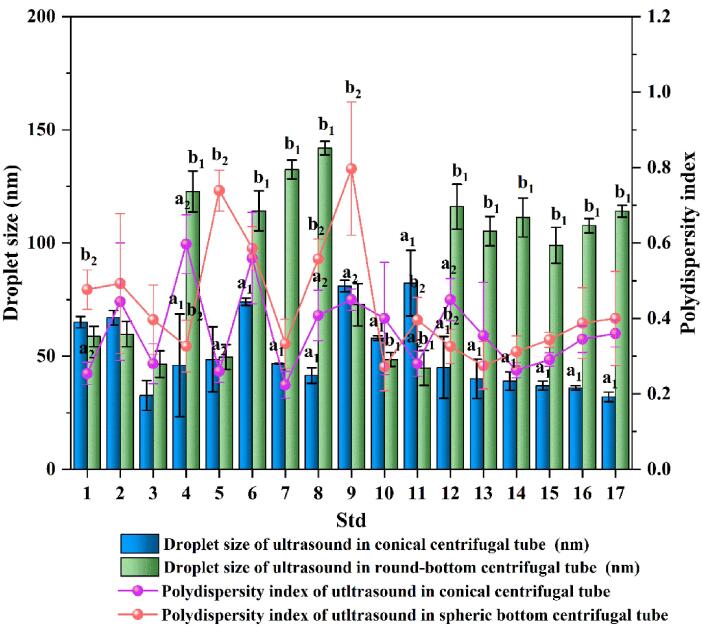
Effects of conical and round-bottom centrifugal tube ultrasonication on mean droplet size and polydispersity index. The data within the same experimental standard deviation indicates a significant difference (*P* < 0.05). *a*_1_ - *b*_1_ and *a*_2_ - *b*_2_ indicate a significant difference for droplet size and PDI, respectively.

Figure 2 demonstrates the 17 groups of droplet size and PDI of 1-mL EO nanoemulsion prepared in conical and round-bottom centrifuge tubes under different sonication distance, sonication amplitude, and sonication time conditions (Table 1) for crude emulsion. Significant differences (*P* < 0.05) were found in the particle size and the PDI of emulsion droplets formed in conical and round-bottom centrifuge tubes for multiple treatment groups of EO crude emulsion.

In this study, the droplet size of EO nanoemulsions ranged from 32.20 to 82.33 nm and the PDI ranged from 0.224 to 0.597 when sonicated in a conical centrifuge tube. When sonicated in a round-bottom centrifuge tube, the droplet size ranged from 46.46 to 141.94 nm and the PDI from 0.272 to 0.797. The results showed that nanometer-sized emulsions were obtained by sonicating the crude emulsion in both vessels with different bottom shapes, but the average droplet size and PDI were smaller in the conical centrifuge tube, indicating that the influence of the vessel geometry on the sonication and sonication in this vessel produced a more effective energy output for the crude emulsion.

Ultrasound produces cavitation by energy input through the ultrasound probe that penetrates deep into the emulsion portion, causing droplet fragmentation and reducing the PDI, thus improving the stability of the nanoemulsion [38]. When ultrasound is performed in a conical centrifuge tube, it is possible that the walls of this vessel are closer to the ultrasound probe and the power reaches the vessel walls with less attenuation, favoring the cavitation of the emulsion at the bottom near the probe with high efficiency. O'Brien [39] concluded that the energy of ultrasonic energy decreased with increasing distance as it propagated through the attenuating material. Khavari *et al.* [40] found that during ultrasound, the pressure gradually decreased with the farther location of the solution from the ultrasound probe. Nanzai *et al.* [41] investigated the influence of vessel diameter on sonochemical efficiency and cavitation dynamics. Vessels with a diameter greater than 90 mm have been shown to exhibit a reduction in sonochemical activity for a 60-mm diameter transducer [41]. The PDI value usually indicates the uniformity and stability of the droplet particle size distribution in emulsions and takes values between 0 and 1 [42]. When the PDI is greater than 0.5, the system is considered to have a broad size distribution, which is not conducive to emulsion stability [43]. In this study, ultrasonic comparison in two containers revealed that conical centrifuge tubes produced smaller droplet sizes and a narrower PDI. Therefore, the conical centrifuge tube was chosen as the sonication vessel for the subsequent study.

### 3.2 Optimization of EONE preparation conditions by RSM

Mathematical models were developed for the Fig. 2 result using Design expert, and ANOVA was performed. Table 2 shows the coefficient of multiple determinations (*R*^2^) and the adjusted coefficient of multiple determinations (adjusted *R*^2^) for droplet size and PDI, indicating the suitability of the regression model. Also, ANOVA was performed to determine the significance of the coefficients of the quadratic polynomial equation. A large *F* value of this term and a small *P* value indicated a significant effect of this term. The mathematical model of droplet size had an *F* value of 30.14 and a *P* value of < 0.0001, indicating the validity of the model. The *X*_1_, *X*_2_, *X*_3_, *X*_1_*X*_3_, *X*_2_^2^, and *X*_3_^2^ model terms in the droplet size equation were significant (*P* < 0.05), while the rest of the terms were insignificant (*P* > 0.05). In addition, the *P* value of the misfit term was 0.1957, which was not significant, indicating that the equations were relatively well simulated and could be well analyzed. The *R*^2^ values and adjusted *R*^2^ of 0.9748 and 0.9425, respectively, indicated that the quadratic polynomial model fit the data well under experimental conditions, and the adjusted *R*^2^ values indicated good agreement between the predicted and experimental values of the model for droplet size (Table 2). The *F* and *P* values of the PDI mathematical model were 16.44 and 0.0006, respectively, indicating the validity of the model. The test results of the PDI showed that the correlation coefficients of *X*_1_, *X*_2_, *X*_3_, *X*_2_*X*_3_, and *X*_2_^2^ were significantly different (*P* < 0.05); the *F* and *P* values of the misfit term were 0.1914 and 0.8972, respectively, and the *R*^2^ and adjusted *R*^2^ values were 0.8819 and 0.8546, respectively, implying that the model had some reference value.

**Table 2:** Analysis of variance of the fitted quadratic equation for droplet size and polydispersity index of nanoemulsions.

Variable	Droplet size (*Y*_1_)		Polydispersity index (*Y*_2_)			
	Sum of squares	*F* value	*P* value	Sum of squares	*F* value	*P* value
Model	4356.70	30.14	<0.0001	0.1812	16.44	0.0006
*X*_1_	157.92	9.83	0.0165	0.1228	100.29	<0.0001
*X*_2_	1614.44	100.53	<0.0001	0.0112	9.10	0.0195
*X*_3_	264.92	16.50	0.0048	0.0119	9.68	0.0170
*X*_1_*X*_2_	32	1.99	0.2009	0.0040	3.24	0.1149
*X*_1_*X*_3_	235.37	14.66	0.0065	0.0034	2.75	0.1414
*X*_2_*X*_3_	51.26	3.20	0.1169	0.0121	9.88	0.0163
*X*_1_^2^	4.07	0.2537	0.6299	0.0016	1.33	0.2860
*X*_2_^2^	933.26	58.12	0.0001	0.0113	9.19	0.0191
*X*_3_^2^	934.20	58.17	0.0001	0.0018	1.47	0.2642
Lack of fit	73.61	2.53	0.1957	0.0011	0.1914	0.8972
*R*^2^	0.9748			0.9548		
Adjusted *R*^2^	0.9425			0.8967		
*Y*_1_ = 36.8 + 4.44*X*_1_ − 14.21*X*_2_ − 5.75*X*_3_ + 0.9838*X*_1_^2^ + 14.89*X*_2_^2^ + 14.90*X*_3_^2^ + 2.83*X*_1_*X*_2_ − 7.67*X*_1_*X*_3_ − 3.58*X*_2_*X*_3_						
*Y*_2_ = 0.3226 + 0.1239*X*_1_ + 0.0373*X*_2_ − 0.0385*X*_3_ + 0.0197*X*_1_^2^ + 0.0517*X*_2_^2^ + 0.0207*X*_3_^2^ + 0.0315*X*_1_*X*_2_ − 0.0290*X*_1_*X*_3_ + 0.055*X*_2_*X*_3_						

The second-order polynomial model equations and regression coefficients of droplet size and PDI are shown in Table 2. The droplet size of EONE decreased gradually as the sonication distance (horizontal distance from the ultrasonic probe to the container wall) decreased and the sonication amplitude and sonication time increased. The increase in sonication distance (horizontal distance from the ultrasonic probe to the container wall) and sonication amplitude, as well as the decrease in sonication time, led to the increase in the PDI.

**Fig. 3:**
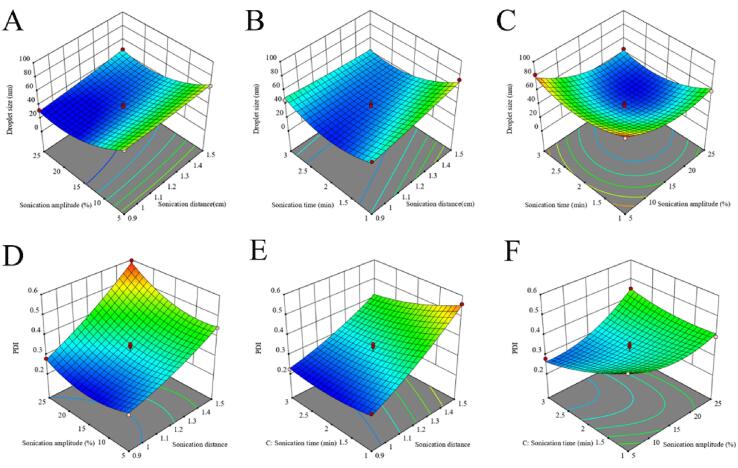
Response surface plots showing combined effects of sonication distance, sonication amplitude, and sonication time on the droplet size (A – C) and PDI (D – F).

Figure 3 shows the 3D response surface and contour plots of the quadratic polynomial model, which presented the effects of the independent variables and their interactions on the droplet particle size and PDI. With constant sonication time, the droplet size of EONE decreased with decreasing sonication distance and increasing sonication amplitude (Fig. 3A). The particle size increased with decreasing sonication time and increasing sonication distance (horizontal distance from the ultrasonic probe to the container wall) with constant sonication amplitude (Fig. 3B). When the sonication distance (horizontal distance from the ultrasonic probe to the container wall) was fixed, the effect of sonication time on the droplet size was small at low amplitude, but the size decreased and then increased with the increase in sonication amplitude; also, the droplet size decreased when the sonication amplitude was higher, and the ultrasonic time increased (Fig. 3C). The PDI increased with the increase in sonication distance (horizontal distance from the ultrasonic probe to the container wall) when the sonication amplitude increased and then decreased with the increase in sonication amplitude when the sonication distance (horizontal distance from the ultrasonic probe to the container wall) was small (Fig. 3D). When the sonication amplitude was constant, the PDI decreased with the decrease in the sonication distance (horizontal distance from ultrasonic probe to container wall) (Fig. 3E). With a fixed sonication distance (horizontal distance from the ultrasonic probe to the container wall), the PDI increased with decreasing sonication time and increasing sonication amplitude (Fig. 3F).

We observed that the particle size and PDI of the emulsion gradually decreased as the distance of the ultrasonic probe from the bottom decreased from 1.5 cm to 0.9 cm (Fig. 3D and 3E). This finding indicated that a more uniform and stronger pressure field was formed below the sonication probe as the distance of the probe in the emulsion increased. Therefore, a more uniform shear stress field was generated in the nanoemulsion system [44]. As the probe penetrated deeper, the acoustic flow generated below the ultrasonic probe was more likely to create effective convection in a limited area of the solution, which was more conducive to achieving more adequate mixing of the nanoemulsion [44].

We also found that the droplet particle size decreased when the sonication amplitude increased. This might be due to more energy input from the probe, which could generate stronger cavitation and shear stress, causing the emulsion to break into smaller droplets [45]. This result was in agreement with the findings of Jesser *et al* [46]. However, when the sonication amplitude was too high, the droplet size increased due to the “over-processing” phenomenon resulting from droplet aggregation. This agreed with the results reported by Kentish *et al* [47].

Finally, we noted that the droplet size and PDI decreased with the increase in sonication time. This might be because the droplets received more cavitation as the sonication time increased, resulting in smaller droplets. This was consistent with the results of Ahmed *et al.*, who concluded that the droplet size of nanoemulsions decreased with increasing preparation time [27]. Based on the target expectations for the EONE droplet size and PDI, we obtained the optimal process parameters: sonication distance of 0.9 cm, sonication amplitude of 18%, and sonication time of 120 s. Under these conditions, EONE emulsions with the smaller droplet size and PDI were predicted.

### 3.3 Particle size, PDI, potential, and encapsulation rate of EONE

**Fig. 4:**
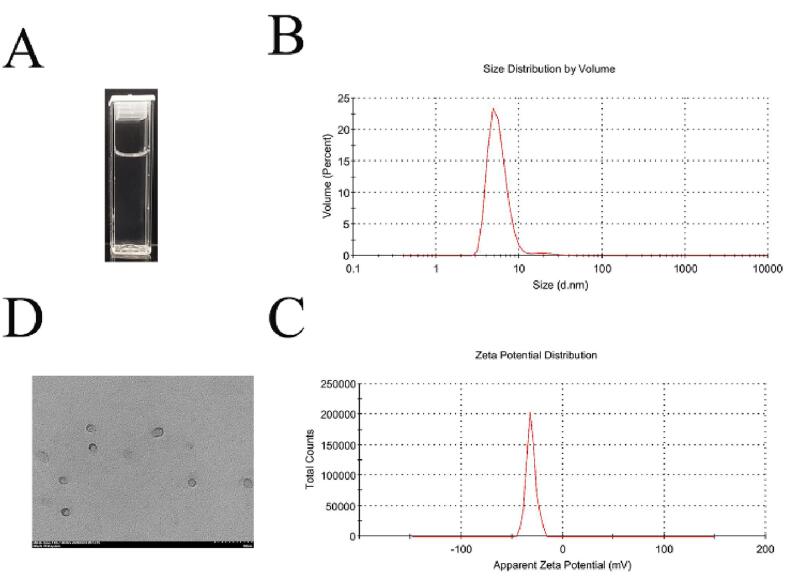
(A) Visual appearance, (B) size distribution, (C) zeta potential distribution, and (D) TEM of EONE.

As shown in Fig. 4, EONE was a transparent liquid with an average droplet size of 18.96 ± 4.66 nm, a PDI of 0.39 ± 0.09, a zeta potential of − 31.17 ± 2.15 mV, and an encapsulation rate of 97.2% ± 1.24%. The droplets were uniformly distributed and spherical in shape under the microscope.

### 3.4 Particle size, zeta potential, and microstructural changes of EONE in each digestion stage

**Fig. 5:**
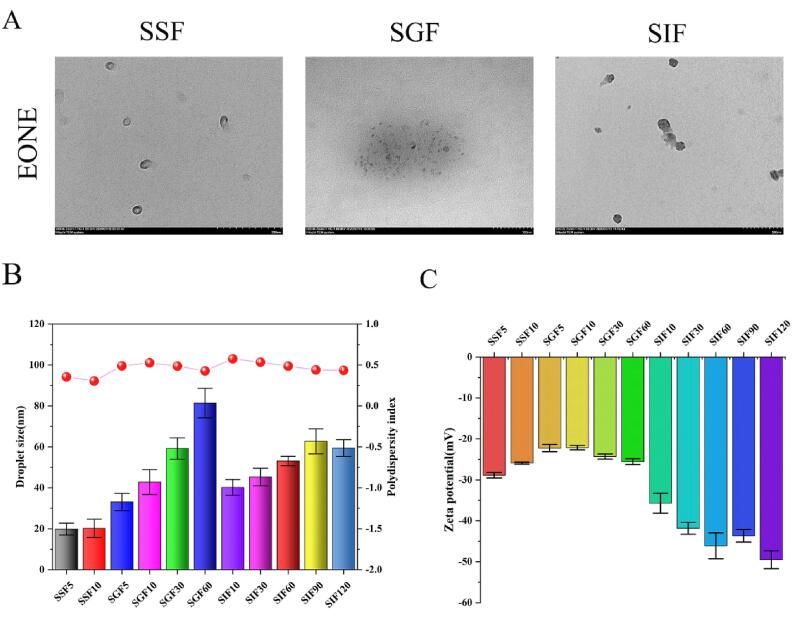
TEM images of EONE during *in vitro* simulated digestion (A). Droplet size and PDI of EONE during SSF incubation for 10 min, SGF incubation for 60 min, and SIF incubation for 120 min (B). Zeta potential values of EONE during SSF incubation for 10 min, SGF incubation for 60 min, and SIF incubation for 120 min (C).

During *in vitro* simulated digestion, changes in the average particle size of the sample were used as an indicator of sample stability. Changes in the interfacial composition of the emulsion may lead to agglomeration or flocculation of droplets and the generation of large droplets. Hence, the microstructural changes in the emulsion were observed using TEM. The changes in EONE are shown in Fig. 5A–5C. During the simulated oral digestion phase, the particle size of EONE did not change significantly (*P* ˃ 0.05) within 10 min, while the absolute value of zeta potential decreased. During the simulated gastric digestion phase, the droplet size of EONE samples increased with the digestion time and reached 81.4 ± 7.2 nm at the end of the digestion, while the absolute value of zeta potential first decreased and then increased. The TEM images showed that the EONE droplets were clustered in large numbers, but the droplets kept a certain distance from each other and did not merge to form large droplets. However, with the increase in incubation time, the droplet size increased, some droplets merged, and oil droplets were observed under the TEM. The EONE absolute value of zeta potential increased continuously during the SIF stage and was 35.7 ± 2.43 mV after 10 min of SIF incubation, which was higher than the initial potential value (*P* < 0.05).

Tween 80 is an efficient nonionic emulsifier with large hydrophilic polyoxyethylene groups, which can produce relatively strong spatial repulsion and make EONE relatively stable in the oral digestive fluid. However, the significant increase in EONE particle size and emulsion aggregation during the SGF phase may be due to the small pH of gastric juice. The large amount of H^+^ in the acidic environment can neutralize the negative charge on the droplet surface, resulting in a lower charge on the droplet surface and a lower electrostatic barrier effect [48]. This suggests that the digestion products of EONE in the gastric juice digestion phase can maintain some surface tension to retain the emulsion morphology but may lack sufficient electrostatic barrier effect to maintain the stability of the spatial structure [49]. However, the enzymes and acidic environment in the stomach have less effect on the emulsification of Tween 80, and thus the droplets mainly aggregate and merge less. Nik *et al.* [50] showed that emulsions prepared with nonionic emulsifiers were stable in acidic gastric fluid. After entering the SIF phase, the pH of the intestinal fluid returns to about neutral, the droplet surface charge increases, an electrostatic barrier effect occurs, the droplets are dispersed, and the droplet particle size decreases. However, the intestinal fluid contains enzymes with strong digestive power, which can disrupt the emulsification and cause the merging of droplets.

Zeta potential measurement was used to determine the interfacial properties of EONE before and after digestion. Due to the electrostatic screening effect, the electrostatic potential depends on the ionic composition of the surrounding medium and usually decreases with the increase in the ionic strength of the aqueous phase [51]. As shown in Fig. 4C, the initial zeta potential value of EONE nanoemulsion was –31.17 ± 2.15 mV. In the SSF stage, the absolute value of the zeta potential of the droplet was reduced due to the effect of salt on electrostatic shielding and ion binding. When entering the SGF phase, the pH around the droplet decreased, and the higher ionic strength and the presence of salt in the simulated gastric juice led to a weakening of the electrostatic force and a decrease in the negative potential of the emulsion [48], [52]. The potential change from SGF10 (–22.13 ± 0.55 mV) to SGF60 (–25.53 ± 0.71 mV) might be caused by the change of particle size of EONE. This reduction in electrostatic repulsive force was also responsible for the aggregation of the sample in the gastric juice, explaining the phenomenon shown in Fig. 5A. The increase in pH in the system in the SIF phase caused the emulsifier to carry more negative charges, and the bile salts might adsorb on the droplet surface or replace hydrolyzed protein material at the interface, leading to an increase in negative charge. Taha *et al.* [53] found that the absolute value of zeta potential decreased as the salt concentration increased in all protein-stabilized emulsions. The potential change in Tween 20/Orange oil nanoemulsion prepared by Qian *et al.* [54] during simulated digestion was consistent with our research results. Ling *et al.* [48] found that the absolute value of the zeta potential of protein nanoemulsions increased in the presence of pepsin, trypsin, and bile salts in the system. This was similar to the results of our study.

### 3.5 Antibacterial activity of EONE in each digestion stage

#### 3.5.1 MIC, MBC, and bactericidal kinetics of EONE against *E. coli* in each digestion stage

**Fig. 6:**
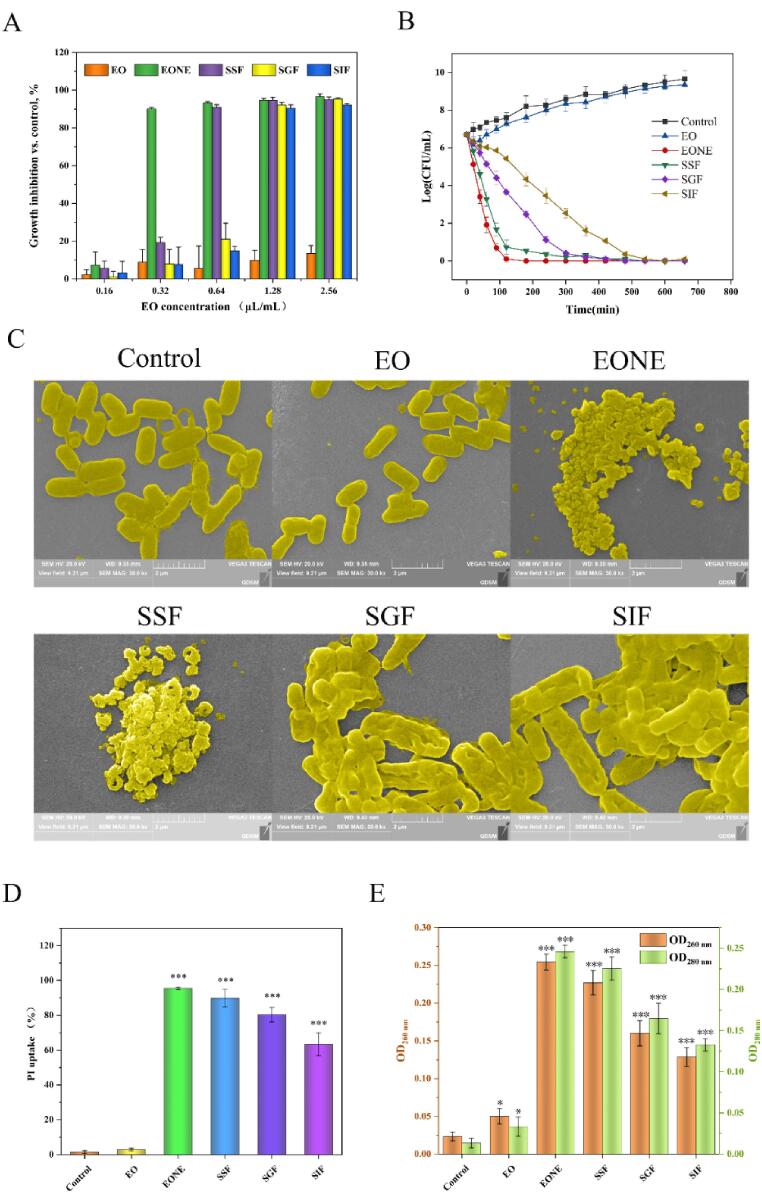
(A) Growth inhibitory activity of EO, EONE, SSF, SGF, and SIF toward *E. coli*. (B) Kinetics of antimicrobial activity of EO, EONE, SSF, SGF, and SIF against *E. coli*. The administrated concentration of various agents was 2.56 μg/mL. (C) SEM images of *E. coli* after 12-h incubation with different treatments. The administrated concentration of various agents was 2.56 μg/mL. (D) Cell membrane integrity of *E. coli*. The administrated concentration of various agents was 2.56 μg/mL. (E) Leakage of intracellular constituents from bacteria after different treatments. The administrated concentration of various agents was 2.56 μg/mL. SSF: EONE during SSF incubation for 10 min; SGF: EONE during SSF incubation for 10 min and SGF for 60 min; SIF: EONE during SSF incubation for 10 min, SGF for 60 min, and SIF for 120 min. *P < 0.05, ***P < 0.001.

The anti-*E. coli* effect of EONE during digestion is shown in Table 3 and Fig. 6A. The results suggested that the ability of EONE to inhibit *E. coli* was affected by the digestion solution, but was better than that of pure EO. The change in the *E. coli* colony number with time under the effect of each antimicrobial agent was studied by the colony counting method. As shown in Fig. 6B, the bacteria in the control and EO groups were in the growth phase from 0 to 11 h, indicating that EO had no bactericidal effect. Compared with the control and EO groups, the number of viable bacteria in the remaining experimental groups decreased significantly. Within 0 – 11 h, EONE, SSF, SGF, and SIF were able to kill 99.9% of the initial number of bacteria in a time-dependent manner, with the number of live bacteria decreasing more significantly with increasing treatment time. EONE was affected by the digestive solution but still had a good bactericidal effect compared with EO.

**Table 3:** MIC and MBC of EO, EONE, SSF, SGF, and SIF for *E. coli.*

Sample	*E. coli*	
	MIC (μL/mL of oil)	MBC (μL/mL of oil)
EO	ND	ND
EONE	0.32	0.64
SSF	0.64	1.28
SGF	1.28	2.56
SIF	1.28	2.56
ND: not detected at the tested concentration.

MIC and MBC are important indicators of the antimicrobial effect of antimicrobial agents; the lower their values, the stronger the antimicrobial ability of antimicrobial agents. Moghimi *et al.* [36] prepared a nanoemulsion from the essential oil of *Thymus vulgaris*, whose antibacterial effect was more than 10 times that of the pure essential oil, which was consistent with the results of the present study. This might be due to the following reasons: (1) Tween 80, as the most commonly used nonionic emulsifier, not only reduced the droplet particle size but also increased the fluidity of the membrane, thus enhancing the bacterial inhibition of EO [55]; and (2) preparing the nanoemulsion increased water solubility and physical stability, which allowed the hydrophobic molecules of the essential oil to have a larger contact area with the surface of the *E. coli* cell membrane.

Nanoscale droplets can increase the passive cellular uptake mechanism, overcome the low permeability of bacterial membranes, and enhance the interaction with bacterial membranes as well as bacterial uptake and intracellular transport due to the low resistance of cell membranes to nanoscale mass transport, thus allowing the disruption of membrane and leakage of cellular components [28], [56], [57], [58].

The thin peptidoglycan layer in the outer membrane of Gram-negative bacteria allows the easier penetration of nanoemulsion droplets and promotes cellular damage [59]. The decrease in bacterial inhibition in the SGF and SIF groups might be due to the aggregation of EONE in the gastrointestinal tract and the increase in particle size, which affected the bacterial inhibitory effect of EONE. The inhibition results showed that EONE retained good antibacterial activity after a short digestion treatment, indicating that EONE alleviated the degradation of digestive juices and facilitated its anti-*E. coli* effect *in vivo*.

#### 3.5.2 Microscopic morphology of *E. Coli* treated with EONE in each digestion stage

The effect of EONE on the microstructure of *E. coli* in the digestion treatment stage was observed using SEM (Fig. 6C). A normal bacterium was rod shaped with a smooth surface of uniform size and full and complete morphology. The EO-treated bacterium showed splitting, and a small part of it showed folds. In the EONE and SSF groups, the structure of the bacterium was collapsed and crumpled, and the bacterium had a large number of cell fragments. The morphology of *E. coli* in the SGF and SIF groups showed irregular deformation, and a large number of groove-like undulating folds appeared on the surface of bacteria.

The EO produced an inhibitory effect by affecting the cell membrane of the bacterium. The disruption of membrane function further affected the metabolic processes of the bacterium [60]. The cells began to swell, permeability increased, and intracellular components outflowed, causing the collapse and cleavage of the bacteriophage cell membrane surface [61]. Oussalah *et al.* [62] studied the inactivation mechanism of Corydohyus capatus oil, Cinnamomum cassia oil, and Satureja montana oil against pathogenic *E. coli* O157:H7 and found that the exposure of *E. coli* cells to the three essential oils resulted in the loss of intracellular components and homeostatic imbalance. Bhargava *et al.* [63] found massive surface disintegration of bacteria exposed to *Corydohyus capatus* oil nanoemulsion. Bajpai *et al.* [61] found that the main components of the essential oil caused damage to the structure and function of cell membranes and produced cellular debris. Moghimi *et al.* [28] showed that the action of the nanosized essential oil on *E. coli* resulted in further disruption and alteration of the cellular morphology of the bacterium. The results of the present study were consistent with these findings, and EONE still had anti-*E. coli* activity after *in vitro* simulated digestion.

#### 3.5.3 Cell membrane integrity assay

PI is a nucleic acid dye that is not cell membrane permeable and can enter bacteria with broken cell membranes, thus distinguishing between living and dead cells [64]. As shown in Fig. 6D, the PI uptake rate increased from 1.49% to 2.82% in the EO group after 2 h treatment, but from 1.49% to 95.49% in the EONE group, to 89.86% in the SSF group, to 80.32% in the SGF group, and to 60.32% in the SIF group compared with the control group. The results, combined with the findings in Fig. 6C, indicated that EONE after *in vitro* simulated digestion could kill *E. coli* by disrupting the cell membrane. Bacterial nucleic acid leakage results are shown in Fig. 6E; all experimental groups showed a significant release of nucleic acids compared with the control group (*P* < 0.05), implying that EONE after digestion led to the release of bacterial intracellular components and promoted bacterial death; these results were consistent with the findings of PI study.

Essential oils usually interact with the cell membrane of microorganisms, leading to dysfunction of the cell membrane. This results in the leakage of cytoplasm, enzymes, and proteins within the membrane, disrupting the normal metabolic process of the bacterium and leading to its death [64], [65]. The changes in bacterial morphology, as well as cell membrane permeability, show that EONE can still interact with microbial cell membranes after treatment with digestive solution.

## 4 Conclusion

The results of this study showed that sonication in a conical centrifuge tube was more effective than sonication in a round-bottom centrifuge tube for the preparation of EONE. The optimal process for the preparation of EONE was determined by RSM: the sonication distance was 0.9 cm, the sonication amplitude was 18 %, and the sonication time was 120 s. The optimized EONE had a particle size of 18.96 ± 4.66 nm, a PDI of 0.39 ± 0.09, a zeta potential of –31.17 ± 2.15 mV, and an encapsulation rate of 97.2% ± 1.24%. In the *in vitro* simulated digestion experiments, we observed using TEM that EONE remained stable in simulated saliva; aggregation occurred in simulated gastric juice without merging. In the SIF, EONE aggregated and merged, and the PDI and the absolute value of zeta potential decreased. The results of MIC, MBC, and bactericidal curves showed that EONE in all stages of the simulated digestive fluid treatment could effectively inhibit the growth of *E. coli* and had bactericidal effects. SEM revealed that EONE in each digestion stage could cause structural damage to *E. coli* cells and wrinkles on the cell surface. PI uptake and nucleic acid and protein assays demonstrated that EONE in each digestion stage could increase the permeability of the *E. coli* cell membrane, disrupting the integrity of the cell membrane and leading to the leakage of intracellular material. Therefore, the ultrasonically prepared EONE enhanced the anti-*E. coli* activity of EO and showed some stability and good anti-*E. coli* activity in the *in vitro* simulated digest, thus providing some research basis for the *in vivo* antibacterial application of the essential oil.

### CRediT authorship contribution statement

**Ruiteng Song:** Conceptualization, Investigation, Methodology, Writing – original draft. **Yongqi Lin:** Formal analysis, Methodology, Validation, Writing – original draft. **Zhenzhen Li:** Resources, Supervision, Writing – review & editing.

## Declaration of Competing Interest

The authors declare that they have no known competing financial interests or personal relationships that could have appeared to influence the work reported in this paper.
